# The antidepressant properties of ketamine (literature review)

**DOI:** 10.1192/j.eurpsy.2023.1759

**Published:** 2023-07-19

**Authors:** F. Z. Chamsi, H. Abrebak, A. Z. essafi, S. Z. radi, A. el ammouri

**Affiliations:** psychiatry department, CHU tanger, tanger, Morocco

## Abstract

**Introduction:**

Major depression is a common condition. Despite significant advances in psychopharmacology since the 1950s, the onset of action and drug resistance remain therapeutic challenges for traditional antidepressant agents, such as serotonin reuptake blockers. The recent discovery of the rapid antidepressant effect of ketamine, receptor antagonist, has revolutionized research in this field.

**Objectives:**

demonstration of the antidepressant properties of ketamine

**Methods:**

For this purpose, major search engines such as Pubmed, medline, Science Direct, and journals specializing in sociology were contacted, with the introduction of keywords (ketamine-Esketamine-resistant depression) and the selection of literature reviews but also articles deemed relevant for this review.

**Results:**

The initial demonstration of ketamine’s antidepressant effects was gradual, rather unusually in a treatment-resistant patient population. First administered in single doses in studies, ketamine showed a rapid and robust antidepressant effect, but not sustained over time. However, studies of repeated doses, spread over a period of a few weeks, then revealed that it was possible to prolong and even improve the clinical response. It is important to mention that the use of ketamine to treat depression still remains.

In 2000, the first randomized-controlled, double-blind clinical study used a crossover design, in which each participant received two infusions over 40 minutes, alternating between one week and one infusion of ketamine (0. 5 mg/kg) and a placebo infusion. A statistically significant antidepressant effect of ketamine compared to placebo was observed as early as 240 minutes after treatment and reached a maximum after 3 days; of the 8 patients treated with ketamine, 7 had an improvement in their symptoms of at least 30% and 4 of at least 50%

In 2006, Zarate et al. carried out the first replication study of the results obtained by the group from Yale University . In this study, 71% and 29% of the 17 patients who received ketamine achieved a response and remission, respectively ,the significant effect of ketamine was revealed after 110 minutes of treatment and until the end of the 7-day post-infusion follow-up.However, one week later, only 35% of patients had reached the clinical response threshold.

In 2010, Diazgranados et al. published the first study of ketamine treatment for bipolar depression. While the first two studies required patients to take no other psychotropic drugs, patients in this study had to show unresponsiveness to a therapeutic dose of lithium or valproic acid, two agents used in the treatment of bipolar disorder. Again here, 71% of patients who received ketamine achieved a clinical response

**Conclusions:**

Finally, note that the discovery of the antidepressant action of ketamine has opened the door to the search for other molecules targeting the glutamatergic system, which will possibly provide an even greater

**Disclosure of Interest:**

None Declared

